# CenterADNet: Infrared Video Target Detection Based on Central Point Regression

**DOI:** 10.3390/s24061778

**Published:** 2024-03-09

**Authors:** Jiaqi Sun, Ming Wei, Jiarong Wang, Ming Zhu, Huilan Lin, Haitao Nie, Xiaotong Deng

**Affiliations:** 1 Changchun Institute of Optics, Fine Mechanics and Physics, Chinese Academy of Sciences, Changchun 130033, China; sunjiaqi19@mails.ucas.ac.cn (J.S.); weiming19@mails.ucas.ac.cn (M.W.); zhuming@ciomp.ac.cn (M.Z.); linhuilan21@mails.ucas.ac.cn (H.L.); niehaitao@ciomp.ac.cn (H.N.); 2 University of Chinese Academy of Sciences, Beijing 100049, China; 3 State Key Laboratory of Astronautic Dynamics, Xi’an 710043, China; caoshenyi@stu.xjtu.edu.cn

**Keywords:** infrared video target detection, different maps, spatial–temporal attention module

## Abstract

Infrared video target detection is a fundamental technology within infrared warning and tracking systems. In long-distance infrared remote sensing images, targets often manifest as circular spots or even single points. Due to the weak and similar characteristics of the target to the background noise, the intelligent detection of these targets is extremely complex. Existing deep learning-based methods are affected by the downsampling of image features by convolutional neural networks, causing the features of small targets to almost disappear. So, we propose a new infrared video weak-target detection network based on central point regression. We focus on suppressing the image background by fusing the different features between consecutive frames with the original image features to eliminate the background’s influence. We also employ high-resolution feature preservation and incorporate a spatial–temporal attention module into the network to capture as many target features as possible and improve detection accuracy. Our method achieves superior results on the infrared image weak aircraft target detection dataset proposed by the National University of Defense Technology, as well as on the simulated dataset generated based on real-world observation. This demonstrates the efficiency of our approach for detecting weak point targets in infrared continuous images.

## 1. Introduction

Infrared video dim-target detection is one of the key technologies in Infrared Search and Track (IRST) systems [1]. Due to its high sensitivity and interference resistance, infrared detection imaging can promptly capture and track targets, enabling the command center to make comprehensive judgments of target properties. However, due to the high observation angle and wide range of infrared detectors, weak-target imaging often appears as merely a small spot or point within the image, making it highly susceptible to being obscured by intricate backgrounds. Moreover, the imaging of the target is often similar to the infrared image noise [2], leading to potential confusion.

**Figure 1:**
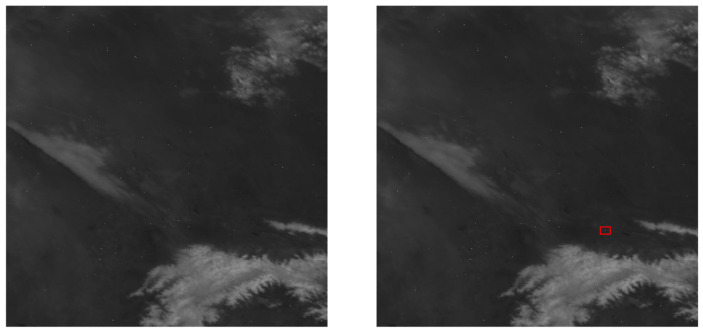
Illustration of consecutive frames in remote infrared long-range infrared remote sensing imaging (dim targets are marked with red boxes in the figure).

Currently, mainstream methods of dim target detection in infrared video still rely on traditional technologies, such as the frame difference method, background modeling, and the optical flow method. However, these methods have many limitations, poor robustness, and require extensive computations when changing application scenarios. With the dawn of the big data era, numerous deep learning-driven approaches have surfaced for detecting faint targets within infrared images [3]. Most of these methods focus on how to utilize feature fusion to obtain more target features while overlooking the significant impact of long-term observations and sequential backgrounds on the detection results of infrared small targets. As shown in Figure 1, in infrared remote sensing images, the target position cannot be determined solely based on a single image. It can only be obtained through the analysis of sequential images. In the case of distant infrared imaging, weak targets are often represented as single point-like objects that are small and dark, with very limited features. Additionally, the background pixels far exceed the target pixels, causing the loss of target features during the downsampling process. Infrareddim-target detection and tracking systems require precise positioning of the target due to the long imaging distance. A deviation of a single pixel can have a significant impact on accurate targeting. Currently, mainstream methods based on bounding box calculations of centroids or centers of mass are not suitable for such application scenarios.

**Figure 2:**
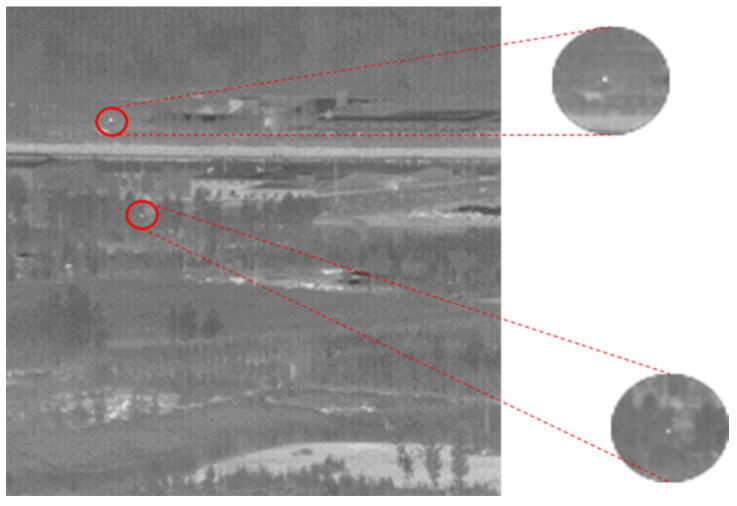
Targets and noise (the upper portion represents noise, while the lower portion represents the target).

In practical applications, background noise has a significant impact on detection performance. The distribution of these noises is random, and even small circular spots caused by minor movements or changes in brightness in the original image can be mistakenly identified as the desired point targets. As shown in Figure 2, these circular noise spots have almost the same features as our target.

To address the aforementioned issues, this paper proposes CenterADNet, a keypoint regression-based method for infrared temporal image target detection. Firstly, this method utilizes keypoint regression as a baseline to realize the accurate positioning of the target. Secondly, to further minimize the impact of background noise, a combination of difference maps and original images is employed to precisely extract features. Additionally, to optimize the use of small-target features, the feature extraction network incorporates a method that preserves high-resolution representations. Additionally, spatiotemporal information is crucial for target characterization. Therefore, a spatiotemporal attention module [4] is integrated into the network for comprehensive target analysis. The main contributions of this paper can be summarized as follows:

- Innovatively incorporating multi-frame difference images into the keypoint regression baseline network to fuse multi-frame features for reducing background influence, and integrating difference image-guided spatiotemporal attention modules into the network structure to fuse multi-frame features.
- For distant infrared imaging with small and dim targets, employing high-resolution preservation methods to extract target features while maximizing the preservation of these features.
- A self-made simulated dataset is created to mimic various forms of infrared point targets in motion. Multiple background abrupt changes are applied to a sequence of images to simulate scenarios where the target flies out of the observation perspective, thus verifying the reliability of the algorithm.

Our algorithm demonstrates superior performance on both the infrared image dataset for weak-aircraft-target detection and tracking [5], proposed by the National University of Defense Technology in 2020, and a simulated dataset generated based on observations of real-world scenarios. These results validate the effectiveness of our algorithm in detecting weak point targets in the infrared domain, surpassing the performance of current mainstream target detection methods.

## 2. Related Work

Due to dynamic changes in a scene, such as weather conditions, variations in lighting, shadows, and interference from cluttered backgrounds, video object detection becomes particularly challenging. One strategy to address this is to enhance the coherence and stability of detection results through post-processing based on temporal information. These methods typically employ object detectors based on static images to obtain detection results, which are then combined. Another approach utilizes temporal information to aggregate features from adjacent frames or entire clips, thereby enhancing the features of the current frame and improving detection performance. This helps to mitigate issues such as motion blur, occlusion, and appearance variations to a certain extent [6]. A third approach involves using optical flow models to estimate the motion of objects and confirm their positions.

Deep learning-based methods for video object detection using static images have been widely researched. For the task of detecting static object in images, Girshick et al. proposed the region CNN (R-CNN) [7]. In their approach, region proposals are extracted using selective search. The candidate regions are then warped to a fixed size and fed into a convolutional neural network (CNN) to extract CNN features. Finally, a support vector machine (SVM) model is trained for object classification. A faster version of R-CNN, called fast R-CNN, is presented in [8]. Compared to the multi-stage pipeline approach of R-CNN, fast R-CNN integrates the object classifier into the network and simultaneously trains the object classifier and bounding box regressor. The introduction of a region-of-interest pooling layer allows for the extraction of fixed-length feature vectors from bounding boxes of different sizes. YOLO [9] (You Only Look Once) and SSD [10] (Single-Shot Multibox Detector) have enhanced the detection efficiency of object detection, aiming to achieve real-time detection. Notably, SSD achieves a mutually beneficial scenario in terms of detection accuracy and efficiency by improving detection efficiency while maintaining detection accuracy. CenterNet [11], representing the anchor-free approach, represents a way to perform object detection without candidate boxes, utilizing keypoint regression for object filtering. The results of static image object detection can be further improved by post-processing based on temporal information, ensuring more coherent and stable detection results in detection.

Video object detection methods are based on inter-frame feature fusion. Relying solely on static object detection does not fully utilize the temporal information provided by video inputs. Inspired by the pioneering work of faster R-CNN [12], a novel approach called temporal CNN (T-CNN) was introduced, which better captures the spatiotemporal information of videos. By incorporating time convolutional networks in [13], the authors normalized the detection results and demonstrated the effectiveness of this approach for video object detection tasks. Building upon faster R-CNN as the backbone, Ref. [14] employed a multi-stage framework to progressively enhance the proposal features of reference frames using proposals from support frames. This method aims to fuse features capturing relationships between multiple proposals. The final advanced-stage features were utilized for classification and regression, with a post-processing method called BLR (Box Linking with Relations) introduced. Ref. [15] utilized LSTM [16] to effectively learn temporal information in video sequences. Their approach combined the static image object detection algorithm SSD with LSTM, fully leveraging the advantages of LSTM to enhance the robustness of video object detection. Ref. [17] combined RNN with the current and neighboring frames’ features, which were fed into the STMM module. STMM aggregates features from previous frames at time t − 1 and the features of the current frame and neighboring frames at time t. The aggregated features are then used for detection. Furthermore, a Transformer [18] simplifies the workflow of video object detection by effectively eliminating many handcrafted feature aggregation components, allowing for end-to-end training.

Deep learning-based object detection algorithms utilize dynamic information such as optical flow [19]. The optical flow model calculates the instantaneous velocities of spatially moving objects on the imaging plane. It utilizes the temporal variations of pixels in an image sequence and the correlation between adjacent frames to determine the corresponding relationships between the previous and current frames, thereby extracting the motion information of objects between neighboring frames. Ref. [20] comprises two modules: optical flow extraction and feature aggregation. The optical flow is extracted using the FlowNet [21] network. For each frame, the optical flow between the current frame and its adjacent frames is computed, and the features of the adjacent frames are combined with the extracted optical flow. Subsequently, in the feature fusion stage, the current feature is combined with multiple neighboring features through element-wise summation. Ref. [22] initially extracted features from frames and used a FlowNet to extract optical flow information between frames. Calibration was performed at the pixel level, followed by instance-level calibration using the predicted instance’s movement, which corresponds to the proposal obtained from R-FCNs (region-based fully convolutional networks). Finally, the features obtained from both pixel-level and instance-level calibration were fused for training and testing. Ref. [23] also started by extracting features from frames and using FlowNet to compute optical flow information between frames. Pixel-level calibration was conducted, followed by instance-level calibration using the predicted instance’s movement (similar to the proposal from R-FCN). The resulting features from both pixel-level and instance-level calibration were then fused for training and testing.

## 3. Methods

### 3.1. Overview

**Figure 3:**
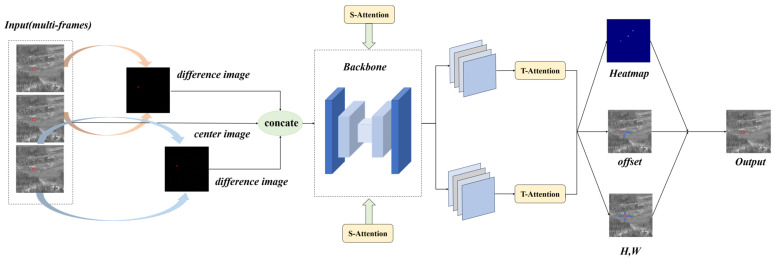
Network-specific flowchart (multiple frames are differentially computed and fed into the network, incorporating attention modules, and ultimately outputting keypoint predictions).

As depicted in Figure 3, our network comprises three main components. In the input stage, we incorporated difference maps generated through frame subtraction as additional input channels (as detailed in Section 3.2). During the feature extraction stage, we enhanced the hourglass [24] backbone network of CenterNet. To address the challenge posed by the low brightness of our point targets, we drew inspiration from an HRNet (high-resolution network) [25] to facilitate continuous high-resolution representation within the hourglass framework. Furthermore, we conducted extensive feature fusion across numerous layers to guarantee the comprehensive extraction of small targets’ features. Notably, guided by difference maps, we integrated spatial and temporal attention modules to bolster the connectivity of targets between frames. Finally, a heatmap was generated through a 1 × 1 convolution. Since our targets are point targets, the resulting keypoint heatmap indicates the target’s location and reflects the intensity at the central position.

### 3.2. Input Layer of Difference Image Data

Compared with single-frame imagery, one of the significant distinctions in video object detection lies in the utilization of temporal information. Leveraging multiple frames can greatly enrich target features and enhance detection performance [26]. While numerous video detection methods primarily focus on multi-frame fusion for target representation, the challenge lies in effectively fusing features for infrared point targets due to their small and dim characteristics. Inspired by the field of video classification TDNs (Temporal Difference Networks) [27], we adopted a different approach, shifting from feature augmentation to background suppression and accentuating the discrepancies between targets and backgrounds to enhance detection performance.

When the camera is fixed, our method is very effective for suppressing background noise. At the initial stage of algorithm verification, we aimed at the simulation image of space-based infrared remote sensing imaging, and the camera angle is fixed. After the target flies out of the field of view, the lens will switch once and then continue to fix the current angle. At this time, we can reduce a lot of background noise by using images subtracted from multiple frames for detection. However, in the case of camera follow-up, there will be slight differences in background between consecutive frames.

So, we explored the strategy of using frame differencing to extract features by jointly inputting the difference images and the original images into the network as distinct channels. Unlike in TDNs, we performed data subtraction during the image input stage rather than feature fusion, thus maximizing the preservation of original data and obtaining more accurate results. Our proposed method is straightforward: assuming the current input image is $I_{i}$, with an input segment length of 2n+1 and $I_{i}$ as the central frame, adjacent input data are subtracted to generate the final dataset {$\left(I_{i-n}-I_{i-n-1}\right),\ldots ,\left(I_{i}-I_{i-1}\right),\left(I_{i}\right),\left(I_{i+1}-I_{i}\right),\ldots ,\left(I_{i+n}-I_{i+n-1}\right)$}. Concatenating these data produces the final input $H_{2n+1}$, where the length of the tensor is equal to the total length of the input segment 2n+1.

Initially, this approach raised doubts because the target of video does not move regularly; the target often moves continuously in the height direction, which is reflected in the two-dimensional image; and the position of the target will hardly change. However, during the method design, we combined the original image as the central channel with the difference map. This allows for the detection of targets even in scenarios where they remain almost static, while the other difference images mostly generate feature maps that are nearly all zeros, minimizing their impact on detection results.

**Figure 4:**
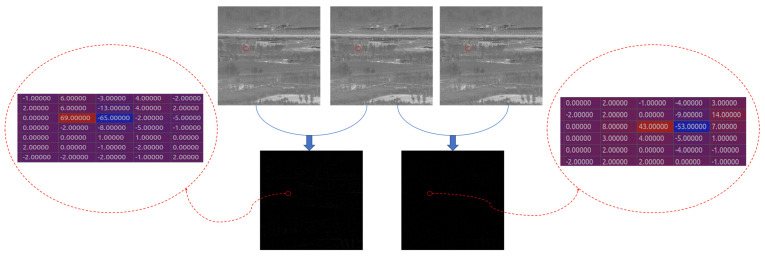
Matrix representation illustrating the results of differential computations across multiple frames.

Figure 4 demonstrates the matrix of differencing consecutive data, highlighting the substantial background suppression achieved by our approach.

### 3.3. Feature Extraction Network and Temporal–Spatial Attention Module

As a classical anchor-free network, CenterNet can predict the keypoints of objects through heatmaps. We chose CenterNet because it does not rely on anchors and only predicts the center points of objects, eliminating the need for post-processing filtering. This makes it highly suitable for our infrared point object detection application.

Given the input images, which consist of multiple frames including difference maps as mentioned in Section 3.2, we define the input image segment length as *n*, the width of the image as w, and the height as h, $I\in R^{w\times h\times n}$. The network output is a downsampled heatmap $\hat{Y}\in {\left[0,1\right]}^{\frac{w}{r}\times \frac{h}{r}\times C}$, where r is the output stride and C is the number of classes (in our case, there is only one class for the observed point object). The keypoint training network adopts focal-loss [28] normalization, dispersing the ground truth keypoints into the heatmap $Y\epsilon {\left[0,1\right]}^{\frac{w}{r}\times \frac{h}{r}\times C}$ using a Gaussian kernel $Y_{x,y,c}=exp(-\frac{{\left(x-{\tilde{\rho }}_{x}\right)}^{2}-{\left(y-{\tilde{\rho }}_{y}\right)}^{2}}{2\sigma_{\rho }^{2}})$, where ρ represents the true coordinates of the keypoints and $\tilde{\rho }\tilde{\;}$ represents their mapped positions on the feature map after network processing. Additionally, the network predicts a local offset (${\hat{O}\in R}^{w\times h\times 2}$), representing the discrete error in object size, through the output stride and a regression (${\hat{S}\in R}^{w\times h\times 2}$).

**Figure 5:**
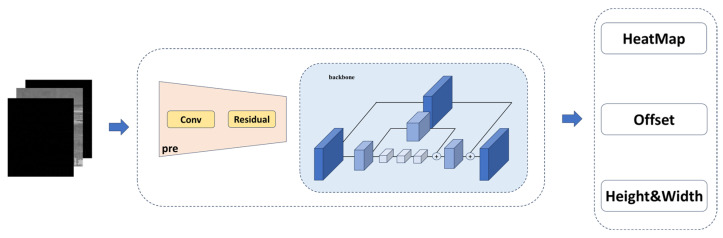
Components of the feature extraction network.

As shown in Figure 5, the entire network consists of three components: A standard convolutional network (in this paper, we utilized the hourglass network to maintain high-resolution representations), a deformable convolutional network built with 3 × 3 deformable convolutional layers (DCLs) [29] and up-convolutional layers, and three heads that output the aforementioned heatmap $\hat{Y}$, keypoint offset ${\hat{O}\in R}^{w\times h\times 2}$, and size offset $\hat{S}$.

During the inference stage, we employed the eight-neighbor comparison method to extract peak points from the heatmap. Let ${\hat{P}}_{c}$ be the set comprising n detected center points, each represented as $\left(x_{i},y_{i}\right)$. The final bounding box was generated as follows:

(1) $$x_{i}+\delta x_{i}-\frac{w_{i}}{2},y_{i}+\delta y_{i}-\frac{h_{i}}{2},x_{i}+\delta x_{i}+w_{i}/2,y_{i}+\delta y_{i}+h_{i}/2$$

where $\left(\delta x_{i},\delta y_{i}\right)={\hat{O}}_{x_{i},y_{i}}$, $\left(w_{i},\;h_{i}\right)={\hat{S}}_{x_{i},y_{i}}$

#### 3.3.1. Feature Extraction Network with Stacked High-Resolution Representation

**Figure 6:**
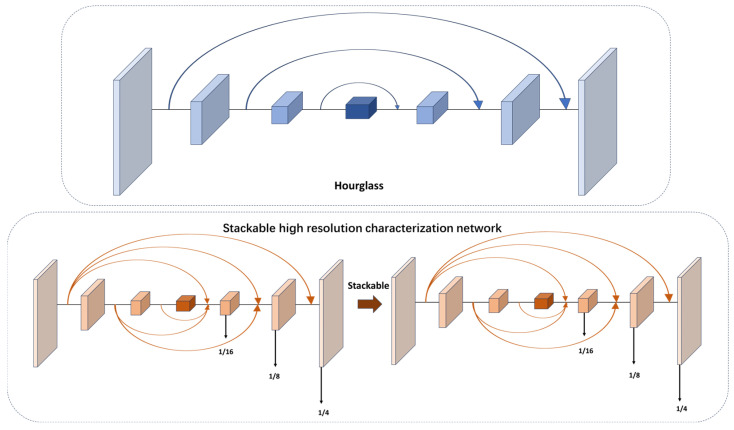
The specific structure of stackable high-resolution network in this paper.

Currently, many feature fusion methods in video target detection are used to fuse the features of different downsampling layers. However, small-target detection is very sensitive to location. It needs continuous high-resolution representation. HRNet [25] introduces the concept of continuous high-resolution representations. In our work, we utilized this idea for the feature extraction of point targets. However, it is important to note that we did not fully adopt the parallel structure of HRNet. Instead, we integrated the continuous high-resolution representation into the densely connected hourglass network. The specific structure of the backbone mentioned earlier is illustrated in Figure 6.

In our proposed backbone, on the basis of hourglass, the upsampling and downsampling of the original recursive mode are improved. It keeps high-resolution features on one branch and downsamples at different multiples on other branches. These downsampled data with different multiples intensively interact, and finally, the original resolution feature map is restored. At the same time, our network was designed as a stackable mode, and the number of stacked layers can be changed by simple parameter setting to test the influence of different stacking multiples on the network performance.

#### 3.3.2. Spatial–Temporal Attention Module

**Figure 7:**
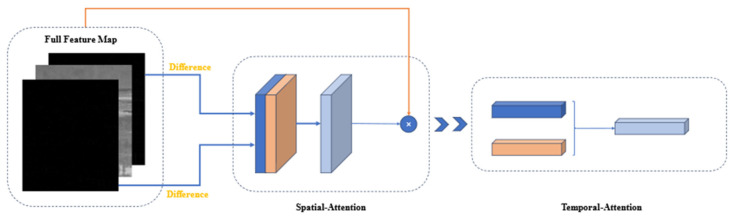
Structural schematic diagram of the spatial–temporal attention module.

We added the attention module [30] to the feature extraction network, as shown in Figure 7.

Spatial attention module: The input features were first passed through the spatial attention module. It is important to note that we did not feed all the data into the spatial attention module. Instead, we focused on the most relevant parts by applying spatial attention to the feature maps generated from the difference maps. This is because we believe that the difference maps eliminate a significant amount of background influence and provide more accurate weights for the target.

The spatial attention module primarily focuses on determining which parts of the features are meaningful. Given an H×W×C feature $F^{i}(i\in \{0,1,\ldots ,I\}$ and I represents the length of the feature containing both the original data and the input difference images), we first extracted the differential feature map $F^{i}$ from $F^{j}$. Then, we applied average pooling and max pooling separately to this differential feature map to obtain two H×W×1 channels. These two channels were concatenated and passed through a 7 × 7 convolutional layer to derive the weight coefficients $M_{s}$. Finally, we multiplied the weight coefficients $M_{s}$ by the full feature map $F^{i}(i\in \{0,1,\ldots ,I\}$ to obtain the scaled new features. The process is presented in the following equation, where ⊗ denotes element-wise multiplication and σ represents the sigmoid function:

(2) $$M_{s}(F^{j})=\sigma \left(f^{7\times 7}\left(\left[AvgPool(F^{j}),MaxPool(F^{j})\right]\right)\right)=\sigma (f^{7\times 7}(\left[F_{avg}^{s},F_{max}^{s}\right]))$$

(3) $${F^{i}}^{\prime }=M_{s}(F^{j})⊗F^{i}$$

Temporal attention module: In our work, the input feature channels contain temporal information about targets. By calculating attention on these channels, we can obtain the combination of spatial and temporal features.

First, we performed global average pooling and max pooling on the spatial dimension to obtain two 1×1×C features. These two channels were then passed through a two-layer neural network (MLP), and the resulting features were summed and passed through a sigmoid function to obtain the weight coefficients $M_{c}$. Finally, we multiplied the weight coefficients by the output of the spatial attention module ${F^{i}}^{\prime }$.

This process can be mathematically represented as follows, where MLP represents the two-layer neural network with the activation function set to ReLU:

(4) $$M_{c}\left({F^{i}}^{\prime }\right)=\sigma (MLP\left(AvgPool\left({F^{i}}^{\prime }\right)\right)+MLP\left(MaxPool\left({F^{i}}^{\prime }\right)\right))$$

(5) $${F^{i}}^{\prime \prime }=M_{c}\left({F^{i}}^{\prime }\right)⊗{F^{i}}^{\prime }$$

In principle, the introduction of the temporal attention module achieves weight allocation for temporal information within the channel, thereby optimizing the representational power of features. By precisely assigning temporal weights, the temporal attention module effectively extracts the temporal features that contribute most significantly to target state changes during the analysis process, serving as the focus of attention in the temporal dimension. Subsequently, these weighted features in the temporal dimension are integrated into their corresponding spatial features, forming a comprehensive, spatial–temporally jointly weighted feature representation. Through this approach, the model can capture and understand the spatial–temporal dynamic behavior of the target more profoundly.

The channel attention module further combines with the spatial attention mechanism to adjust the feature responses at each spatial location accordingly. This collaborative weighted processing of spatial–temporal information not only enhances sensitivity to dynamic targets but also reinforces the network’s ability to ignore background noise and focus on meaningful spatial–temporal patterns.

## 4. Experiment

### 4.1. Dataset and Setup

The Small Aircraft Detection and Tracking Dataset in Ground/Air Infrared Background of the National University of Defense Technology: This dataset is the first comprehensive publicly available dataset specifically designed for detection and recognition purposes in infrared imagery, filling the data gap in the field of infrared object detection and recognition. The dataset consists of 22 data segments collected using unmanned aerial vehicles. Given that our target focuses on the distant infrared imaging of point targets in long-range strike operations, we conducted experiments using only data5, data10, and data21. The details of data 5, data 10 and data 21 are shown in Table 1. These specific datasets feature long-range imaging, single targets, and ground backgrounds. Data5 encompasses long-term observations with a continuous sequence of three thousand frames, while the other two datasets capture short-term observations with only a few hundred frames. All the images are infrared medium-wave imaging with an image size of 256 × 256.

**Figure 8:**
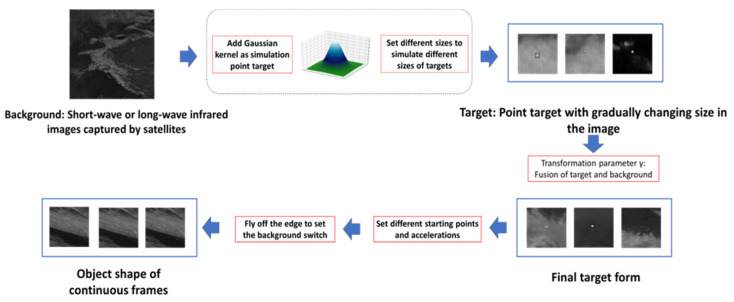
Flowchart of implementation steps of simulation dataset MIRPT.

**Figure 9:**
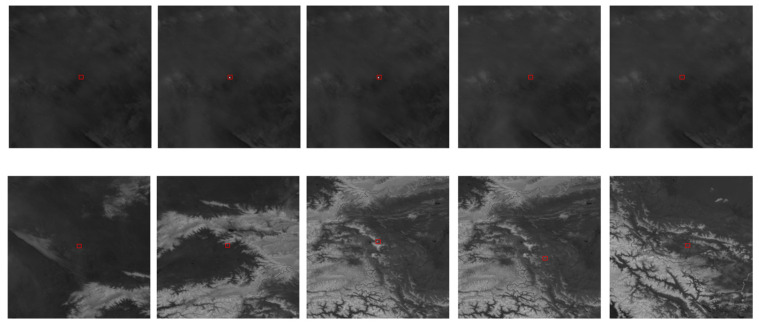
Visualization of a subset of the simulated dataset MIRPT.

Self-made Infrared Point Target Detection and Tracking Simulation Dataset (MIRPT, Multi-frame InfraRed Point Target): As Infrared Search and Track (IRST) systems are mostly applied to military targets, capturing and tracking targets through high-angle satellite observations, there will be changes in target shape and speed during the movement of military targets due to target ignition or stage separation. However, the target size and speed of the infrared image dataset for small aircraft target detection and tracking mentioned above are very stable. Therefore, in order to test the performance of the algorithm when the target’s shape and speed change, we generated a series of simulated datasets by observing real-time satellite observation data of the ground. These datasets simulate distant observations of targets, which manifest as progressively changing point targets in size within the images. Point target simulation mainly depends on Gaussian kernel generation. The targets are fused with the background, simulating various flight trajectories. Figure 8 shows the main flow of adding the simulation target to the background image. The simulated data include targets with different velocities, such as cases where vertical ascent does not result in obvious displacement in the image, as well as targets exhibiting acceleration, deceleration, and sudden shifts in position due to abrupt changes in observation angles. Figure 9 showcases a partial view of the dataset.

There were six data segments in our experiment, among which the infrared image target detection and tracking dataset contains three data segments, and the simulation dataset contains three data segments. Among them, a group of data21 in the infrared image dim and small aircraft target detection and tracking dataset was used as separate test data to verify the robustness of the algorithm in different backgrounds. The other two groups were divided into 2800 training sets and 600 verification sets in proportion. The three sets of data of MIRPT include 5120 training sets and 1020 verification sets. Our network training was conducted using the PyTorch framework on an NVIDIA GTX 2080ti GPU. The experimental settings included a batch size of 16 and 20 epochs. We employed the Adam optimizer with an initial learning rate of 0.01, which decayed by a factor of $10^{-1}$ every 30 epochs. The network parameters and evaluation metrics were referenced from CenterNet.

GT: GT is the abbreviation of ground truth, which is the verified real label provided in the dataset.

TP: the number of detection boxes with IoU > 0.5 (counted only once for each ground truth).

FP: The number of detection boxes with IoU ≤ 0.5 or redundant detection boxes for the same ground truth. AP (average precision): area under the precision–recall (PR) curve.

AR (average recall): The maximum recall of detecting a fixed number of detections per image. In our experiments, the specific value chosen was maxDets = 10.

mAP (mean average precision): this represents the average AP across different categories. Since we only have one category, mAP is equivalent to AP in our case.

Among them, TN and FN are generally not mentioned, because negative samples are not displayed, and there is no problem of distinguishing between true and false. IoU is the abbreviation of Intersection over Union, also known as the overlapping joint ratio. It is a measure used to evaluate the degree of overlap between the bounding box and the real labeling box in a target detection task. IoU measures their overlapping degree by calculating the ratio between the intersection area between the prediction box and the real annotation box and their union area. The value range of IoU is between 0 and 1, and the specific calculation formula is as follows:

IoU = (intersection area)/(area of predicted bounding box + area of real dimension bounding box-intersection area)

TP and FP were used to evaluate each algorithm in our experiment.

(6) $$precision=\frac{TP}{TP+FP}$$

(7) $$recall=\frac{TP}{GT}$$

In addition, we devised a novel evaluation metric. As our objective involved point target localization, the precise positioning of target keypoints became more crucial than detecting bounding boxes. The new evaluation metric replaced IoU with distance calculation between predicted keypoints and ground truth keypoints. A threshold of 1 was set in this evaluation, yielding more accurate results. In subsequent experiments, we will evaluate the detection performance using both evaluation metrics.

### 4.2. Ablation Study

#### 4.2.1. The Combination Performance of the Model

We compared our proposed network model with the original baseline network and validated that our model exhibits significant improvements in infrared point target detection. To assess the effectiveness of the strategies we proposed in enhancing detection performance, we added or removed these modules on top of the baseline network to evaluate the role and importance of each component. The experimental results are presented in the table below (the experimental data present the average test results of the two mentioned datasets in Section 4.1).

Table 2 demonstrates that all three strategies we proposed resulted in improved multi-frame detection accuracy for the two infrared point target datasets. Compared with the calculation results of IoU, the evaluation index based on the distance between the center point was slightly decreased. This is due to the stricter restriction imposed by the center point threshold of 1 compared to the IoU > 0.5 calculation. Importantly, the addition of multi-frame difference input significantly outperformed the use of CenterNet alone, demonstrating the effectiveness of the strategies applied to infrared multi-frame point target detection. The incorporation of the improved high-resolution stacked hourglass feature extraction network effectively extracted features for small and weak point targets. Additionally, the temporal–spatial attention module leveraged the information between consecutive frames, leading to improved detection performance.

#### 4.2.2. Multi-Frame Difference Map Input

In Table 3, we present the specific performance improvements of the network when incorporating the multi-frame difference images as channel inputs (as proposed in Section 3.2). We compared the differences between the original data input and the inclusion of different numbers of difference maps into the network. The results demonstrate that our multi-frame difference image input strategy is effective for multi-frame consecutive object detection. However, as the number of difference map inputs increases, the performance of the network reaches a saturated state. It is crucial to set a highly reasonable and effective duration for the data segments. Through our experimental validation, we found that controlling the number of difference maps at three is a reasonable approach for our dataset. While using more difference map inputs may result in some performance changes, it significantly increases the computational burden of the network, making it an impractical solution.

#### 4.2.3. Effect of Stacked High-Resolution Feature Extraction Network

Table 4 presents the performance of our proposed feature extraction network. We compared the experimental performance of the original hourglass feature extraction network with our newly proposed stacked high-resolution hourglass. Additionally, we conducted a detailed experimental validation by modifying the number of stacked layers and the downsampling factor of the network’s feature extraction process.

Different numbers of stacked layers and downsampling factors had varying impacts on the network’s performance. Through the experiments, we discovered that more stacked layers in the feature extraction network did not necessarily lead to better performance. This result may be attributed to the accumulation of errors during the stacking process, gradually weakening the features of small and weak point targets.

#### 4.2.4. Effect of TSAM (Temporal–Spatial Attention Module)

In this section, we investigated the impact of incorporating TSAM (temporal–spatial attention module) into the network on the network’s performance. Enhancing network performance by integrating attention modules into the network is a commonly used approach. The inclusion of attention modules aims to examine whether the attention mechanism has a noticeable effect on our specific objectives. As shown in Table 5, it is evident that the incorporation of attention modules has a positive impact on both single-frame and multi-frame detection. Our TSAM is an improvement based on CSAM (Channel–Spatial Attention), and we validated the effectiveness of combining CenterNet and CSAM in single-frame detection. Since our data consist of consecutive multi-frame data, temporal information can be utilized. Our TSAM incorporated temporal information into the attention module, leading to a partial improvement in detection performance, as indicated by the data in Table 5.

### 4.3. Comparison with Other Static Detectors

In this section, we present a comparative analysis of our proposed method with other detectors. We evaluate the performance differences between our network and other classic or state-of-the-art object detectors. These methods, both classic and relatively new, include the YOLO series (YOLOv7 [31], YOLOv8 [32]), Faster R-CNN [8], Cascade R-CNN [33], and infrared-dim and small-target detector with excellent performance. It is worth noting that YOLOv8, similar to our algorithm, is an anchor-free approach. The table below illustrates the comparison between our algorithm, CenterADNet, which incorporates multiple strategic integrations, and these classic and newer object detection algorithms.

**Figure 10:**
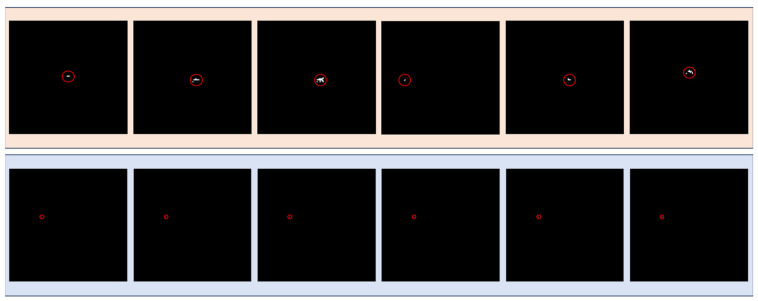
Mask image of MIRST (first line) and mask image of infrared-weak aircraft dataset (second line).

It should be mentioned that LESPS [34] and iSmallnet [35] need to utilize mask data for training. Our simulated dataset can generate masks independently. Since the infrared small aircraft dataset we used does not have mask annotations, we designed a 3 × 3 bounding box based on the given point target location coordinates to select the target, and then performed threshold segmentation on the local area. After processing, manual verification was conducted. We annotated a total of 3901 images. The mask images are shown in Figure 10.

From the Table 6, it can be observed that these algorithms demonstrated relatively high detection accuracy on our simulated dataset. This might be attributed to the high quality of our simulated data, where the targets are comparatively clear. Our baseline algorithm, CenterNet, also exhibited superior performance compared to these algorithms. The performance of our approach was similar to that of the LESPS algorithm in CVPR2023, and our algorithm performed slightly better than LESPS on the simulated dataset.

The third column in the Table 6 displays a comparison of these algorithms in terms of the number of parameters. It is evident that many excellent algorithms have a significantly lower model complexity compared to our algorithm, and an increase in model complexity can lead to a decrease in computational speed. However, in this paper, to adapt to point targets and use CenterNet to obtain the network’s response to the centrality of point targets, we chose CenterNet as the benchmark network for experiments. As can be seen, our algorithm increased the amount of computation by about 20 M compared to the benchmark network but improved accuracy by more than 5%. Since our algorithm is based on an improvement in CenterNet, the addition of densely connected networks and differential image input layers inevitably led to an increase in the number of parameters and a decrease in inference speed. Nevertheless, in practice, our model can complete calculations for 256 × 256 images in about 1 s during the inference process, making it suitable for most object detection tasks in real-world applications. However, reducing model complexity and integrating lightweight models into hardware remain areas for improvement in our future work.

### 4.4. Other Experiments

**Figure 11:**
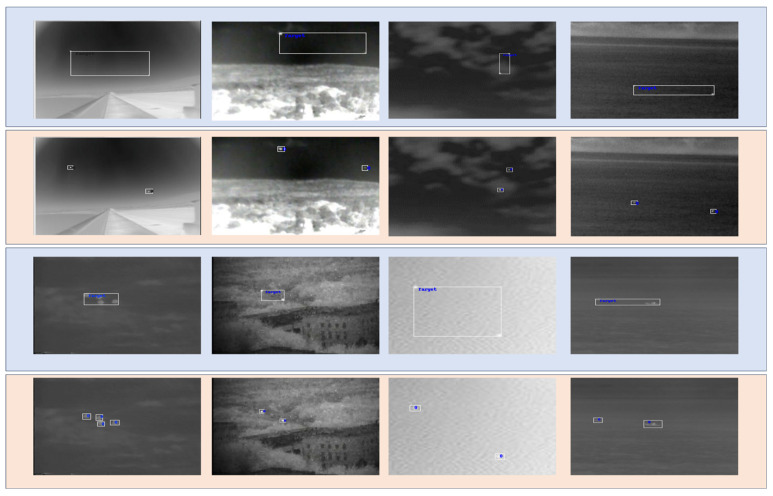
The annotation box of SIRST dataset is partially modified.

In order to compare with the general infrared-weak small-target detection algorithm, we conducted relevant supplementary experiments using the NUAA-SIRST (SIRST) dataset, which is a general dataset for infrared-weak small targets. Due to the fact that most infrared-weak small-target detection is based on target segmentation, the dataset’s bbox annotation files are somewhat lacking. Therefore, we spent some time re-annotating the dataset with bbox annotations, and the specific modifications to the images are shown in the following Figure 11: the blue part represents the original data annotation, while the orange part represents the images re-annotated by us.

The SIRST dataset contains a total of 427 images. We divided the dataset into a training set of 341 images and a test set of 86 images according to the data splitting method used in the original dataset, and conducted relevant experiments. We incorporated our method into the experimental results based on those used in iSmallnet. In the Table 7, the red portion represents the best-performing model, and the bold blue part represents our proposed model. However, since our model requires the use of inter-frame difference images, it does not perform optimally on single-frame small-target detection datasets. Nevertheless, it can be observed that our model achieved performance very close to the optimal model, demonstrating that the network structure we proposed is suitable for feature extraction in the detection of infrared-weak small targets.

### 4.5. Visualization of Results

**Figure 12:**
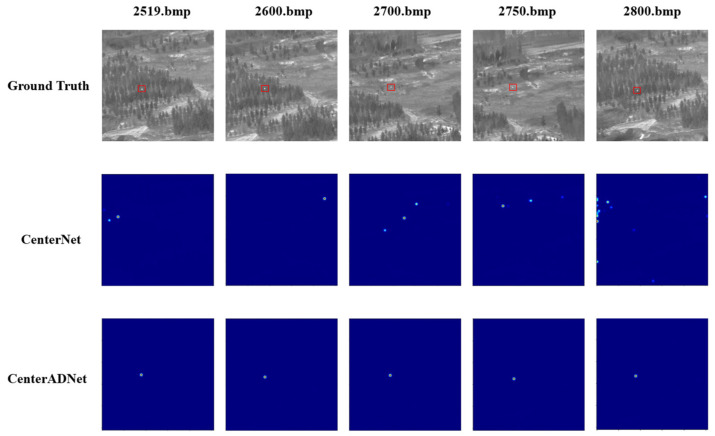
Visualization results of detection keypoints of CenterNet and CenterADNet.

In this section, we present specific visualizations of the detection results achieved by our proposed method. Figure 12 shows the heatmap visualizations of selected data samples. From the images, it is apparent that our proposed method significantly reduced false positives compared to the original approach. The original method still exhibited numerous instances of missed detections, often mistaking the changing speckles between trees as potential targets, leading to a significant decrease in the probability of correctly identifying true targets as keypoints. In contrast, our proposed method exhibited greater stability, although there is a certain degree of positional displacement. However, the majority of these displacements fell within the centroid distance verification metric we proposed.

**Figure 13:**
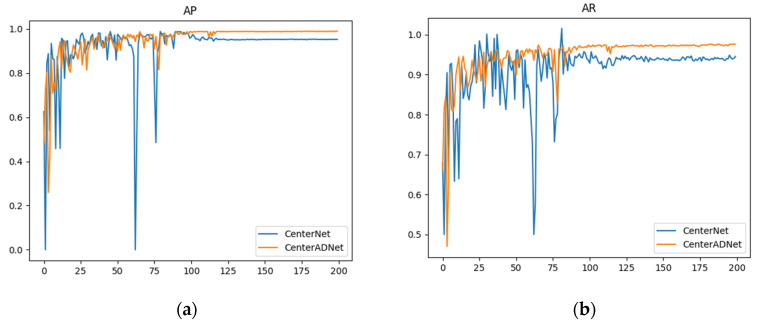
Performance curves of CenterNet and CenterADNet: (**a**) AP performance curve; (**b**) AR performance curve.

Figure 13 illustrates the performance of our method compared to the original approach in terms of accuracy. These experimental results represent the average performance obtained from the five datasets we used. The graph clearly shows that our method not only achieved higher accuracy but also demonstrated greater stability compared to the original algorithm. The original algorithm exhibited some sudden fluctuations in the numerical values. Despite conducting multiple experiments, this issue persisted. We hypothesize that this is because the original detector does not leverage temporal information from multiple frames. As a result, it is susceptible to slight variations in single-frame images, which can significantly impact detection results.

In addition, to validate the robustness of our algorithm, we conducted a separate set of tests on images with a completely new background. The dataset is data21, mentioned in Section 4.1. This set of data contains 500 test images. The target in the picture is still a point target, but the brightness of the target is quite different, and the imaging is blurred. Previous tests involved splitting a complete dataset into training and testing sets, where the backgrounds were relatively consistent, making the detection task less challenging. To assess our algorithm’s performance, we introduced a new set of data with a completely different background.

**Figure 14:**
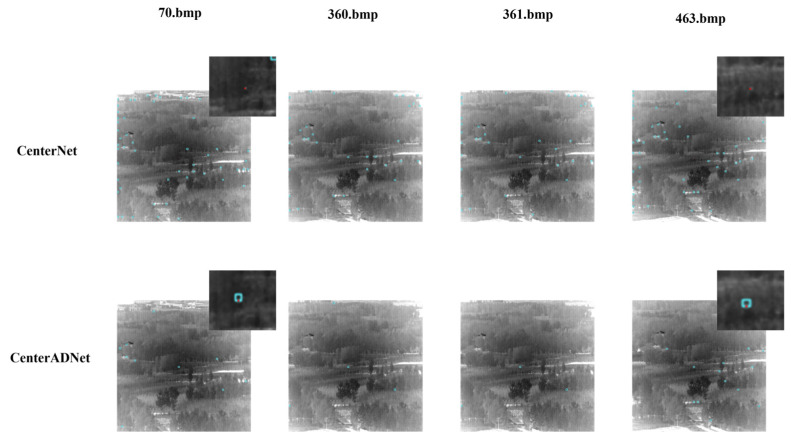
Effect demonstration on another dataset.

Figure 14 showcases the detection results of our algorithm on the images with the new background. It is evident from the graph that both algorithms generate numerous false positives when facing a new background. However, our algorithm significantly reduces the number of false positives compared to the original algorithm. Additionally, the original algorithm tends to lose track of the targets in many frames, while our algorithm demonstrates the ability to track the targets over a longer period.

## 5. Conclusions

In this paper, we proposed a novel algorithm called CenterADNet for infrared video object detection. In our work, we compared several advanced static detectors with our proposed method. We explored various approaches and ultimately adopted center point regression as the foundation of our algorithm. This method can determine the key positions of small and weak targets more accurately, and we further improved this method. The main enhancements include performing multi-frame diffusion on the input data of the algorithm, so as to minimize the influence of background on the detection results by using the similarity of backgrounds in video images. Additionally, we modified the network structure to cater to the characteristics of small and weak targets. We employed a high-resolution feature preservation in the feature extraction network and integrated a spatial–temporal attention module into the network to maximize the utilization of temporal information in video images. Extensive experiments confirmed that our algorithm achieved superior performance in infrared point target video detection. We compared this with baseline methods and visualized the results to demonstrate the outstanding performance and robust anomaly scoring of our algorithm model.

However, our algorithm still has limitations. Despite yielding better results than the baseline algorithm and most static detectors, a few false alarms remain unresolved. Moreover, due to the addition of more input and high-resolution networks, the calculation speed will also decrease to some extent. In addition, infrared point targets encounter numerous complex backgrounds in real-world scenarios, and our dataset cannot cover all possible situations. This highlights the need for continuous data accumulation and algorithm improvement in the future.

## Figures and Tables

**Table 1:** Detailed description of the dataset.

Data	Frames	Scene
Data5	3000	Long-distance, single target, long-term observation
Data10	401	Long-distance, single target
Data21	500	Long-distance, single target

**Table 2:** The specific contribution of the three strategies we proposed to the combined detection accuracy (bold in the table shows the best performance of the algorithm).

Method	AP (IOU)	AR (IOU)	AP (Distance)	AR (Distance)
CenterNet	0.9529	0.945	0.864	0.76
CenterNet + difference maps	0.9746	0.966	0.873	0.79
CenterNet + Stacked High-Resolution Hourglass	0.9552	0.946	0.868	0.77
CenterNet (Multi-Frame) + (STAM)	0.9806	0.968	0.875	0.82
CenterADNet	**0.9895**	**0.974**	**0.878**	**0.82**

**Table 3:** Influence of different difference maps input on detection performance (bold in the table shows the best performance of the algorithm).

Method	AP (IOU)	AR (IOU)	AP (Distance)	AR (Distance)
CenterNet	0.9529	0.945	0.864	0.76
CenterNet + (2 difference images)	**0.9746**	0.966	0.872	0.74
CenterNet + (4 difference images)	0.972	**0.967**	**0.873**	**0.79**
CenterNet + (6 difference images)	0.965	0.957	0.864	0.69

**Table 4:** Influence of different downsampling multiples and stacking multiples on detection performance (bold in the table shows the best performance of the algorithm).

Method		AP (IOU)	AR (IOU)	AP (Distance)	AR (Distance)
CenterNet		0.9529	0.945	0.864	0.76
CenterNet	Stacked (2) + Max Downsample (2)	**0.9552**	**0.946**	0.862	**0.78**
CenterNet	Stacked (3) + Max Downsample (2)	0.9543	0.945	**0.868**	0.77
CenterNet	Stacked (2) + Max Downsample (3)	0.9545	0.945	0.853	0.73
CenterNet	Stacked (2) + Max Downsample (4)	0.9524	0.93	0.855	0.69

**Table 5:** Influence of temporal–spatial attention module (TSAM) on detection performance (bold in the table shows the best performance of the algorithm).

Method	AP (IOU)	AR (IOU)	AP (Distance)	AR (Distance)
CenterNet (Single-Frame)	0.9529	0.945	0.864	0.76
CenterNet (Single-Frame) + CSAM	0.9597	0.952	0.869	0.76
CenterNet (Multi-Frame)	0.9746	0.966	0.873	0.79
CenterNet (Multi-Frame) + TSAM	**0.9806**	**0.968**	**0.875**	**0.82**

**Table 6:** Comparison with other advanced static detectors (bold in the table shows the best performance of the algorithm).

Method	Reference	Parameters (M)	Small Aircraft Detection and Tracking Dataset		Simulation Dataset (MIRPT)	
			AP (IOU)	AR (IOU)	AP (IOU)	AR (IOU)
YoloV5 [36]	Ultralytics’20	7.2 (YoloV5s)	0.977	0.974	0.983	0.855
YoloV7 [31]	Ultralytics’22	36.49	0.646	0.586	0.964	0.797
YoloV8 [32]	Ultralytics’23	11.2 (YoloV8s)	0.712	0.262	0.971	0.843
faster-RCNN [8]	arXiv’15	278 (VGG)	0.685	0.564	0.969	0.826
cascade-RCNN [33]	arXiv’17	704 (VGG)	0.713	0.589	0.973	0.835
Yolov8-spd [37]	ECML PKDD’22	12.8	0.935	0.881	0.967	0.804
LESPS [34]	CVPR’23	4.7	0.987	0.990	**0.99**	0.932
ISmallnet [35]	arXiv’23	9.69	0.938	0.982	0.963	0.945
CenterNet [11]	CVPR’19	104.8 (Hourglass-52)	0.9258	0.968	0.98	0.922
CenterADNet	OURS	129.46	**0.989**	**0.994**	**0.99**	**0.954**

**Table 7:** Performance of each algorithm on NUAA-SIRST dataset (the red portion represents the best-performing model, and the bold blue part represents our proposed model).

Method	Reference	NUAA-SIRST	
		Precision	Recall
Filtering-Based: Top-Hat [38]	OE’96	67.23	44.47
Filtering-Based: Max-Median [39]	SPIE’99	57.35	60.48
Local Contrast-Based: LCM [40]	TGRS’13	64.23	27.81
Local Contrast-Based: TLLCM [41]	TGRS’19	67.68	31.58
Low Rank-Based: IPI [42]	TIP’13	69.29	64.47
Low Rank-Based: RIPT [43]	J-STARS’17	75.48	69.71
CNN-Based: TBCNet [44]	arXiv’19	78.37	48.76
CNN-Based: MDvsFA-cGAN [45]	ICCV’19	83.47	52.47
CNN-Based: ACMNet [46]	WACV’21	87.58	69.61
CNN-Based: ALCNet [47]	TGRS’21	88.16	56.43
iSmallNet [35]	arXiv’23	88.74	90.79
Yolov8-spd [37]	arXiv’22	88.13	91.82
CenterNet [11]	arXiv’19	86.72	88.33
CenterADnet (part model)	ours	**87.93**	**90.84**

## Data Availability

Data contained within the article.

## References

[1] Liu F., Gao C., Chen F., Meng D., Zuo W., Gao X. (2021). Infrared Small-Dim Target Detection with Transformer under Complex Backgrounds. arXiv.

[2] Aliha A., Liu Y., Ma Y., Hu Y., Pan Z., Zhou G. (2023). A Spatial–Temporal Block-Matching Patch-Tensor Model for Infrared Small Moving Target Detection in Complex Scenes. Remote Sens..

[3] Zhang L., Han P., Xi J., Zuo Z. (2023). Infrared Small Target Detection Based on a Temporally-Aware Fully Convolutional Neural Network. Remote Sens..

[4] Chu Q., Ouyang W., Li H., Wang X., Liu B., Yu N. Online multi-object tracking using CNN-based single object tracker with spatial-temporal attention mechanism. Proceedings of the IEEE International Conference on Computer Vision.

[5] Hui B., Song Z., Fan H., Zhong P., Hu W., Zhang X., Ling J., Su H., Jin W., Zhang Y. (2020). A dataset for infrared detection and tracking of dim-small aircraft targets under ground/air background. China Sci. Data.

[6] He L., Zhou Q., Li X., Niu L., Cheng G., Li X., Liu W., Tong Y., Ma L., Zhang L. End-to-end video object detection with spatial-temporal transformers. Proceedings of the 29th ACM International Conference on Multimedia.

[7] Girshick R., Donahue J., Darrell T., Malik J. Rich Feature Hierarchies for Accurate Object Detection and Semantic Segmentation. Proceedings of the IEEE Conference on Computer Vision and Pattern Recognition.

[8] Ren S., He K., Girshick R., Sun J. (2017). Faster R-CNN: Towards Real-Time Object Detection with Region Proposal Networks. IEEE Trans. Pattern Anal. Mach. Intell..

[9] Redmon J., Divvala S., Girshick R., Farhadi A. You only look once: Unified, real-time object detection. Proceedings of the IEEE Conference on Computer Vision and Pattern Recognition.

[10] Liu W., Anguelov D., Erhan D., Szegedy C., Reed S., Fu C.-Y., Berg A.C. Ssd: Single shot multibox detector. Proceedings of the Computer Vision—ECCV 2016: 14th European Conference.

[11] Duan K., Bai S., Xie L., Qi H., Huang Q., Tian Q. Centernet: Keypoint triplets for object detection. Proceedings of the IEEE/CVF International Conference on Computer Vision.

[12] Hou R., Chen C., Shah M. Tube Convolutional Neural Network (T-CNN) for Action Detection in Videos. Proceedings of the IEEE International Conference on Computer Vision.

[13] Bai S., Kolter J.Z., Koltun V. (2018). An empirical evaluation of generic convolutional and recurrent networks for sequence modeling. arXiv.

[14] Deng J., Pan Y., Yao T., Zhou W., Li H., Mei T. Relation Distillation Networks for Video Object Detection. Proceedings of the 2019 IEEE/CVF International Conference on Computer Vision (ICCV).

[15] Lu Y., Lu C., Tang C.-K. Online video object detection using association LSTM. Proceedings of the IEEE International Conference on Computer Vision.

[16] Gers F.A., Schmidhuber J., Cummins F. (2000). Learning to forget: Continual prediction with LSTM. Neural Comput..

[17] Xiao F., Lee Y.J. Video object detection with an aligned spatial-temporal memory. Proceedings of the European Conference on Computer Vision (ECCV).

[18] Vaswani A., Shazeer N., Parmar N., Uszkoreit J., Jones L., Gomez A.N., Kaiser Ł., Polosukhin I. (2017). Attention is all you need. Adv. Neural Inf. Process. Syst..

[19] Bouguet J.-Y. (2001). Pyramidal implementation of the affine lucas kanade feature tracker description of the algorithm. Intel Corp..

[20] Zhu X., Wang Y., Dai J., Yuan L., Wei Y. Flow-guided feature aggregation for video object detection. Proceedings of the IEEE International Conference on Computer Vision.

[21] Dosovitskiy A., Fischer P., Ilg E., Hausser P., Hazirbas C., Golkov V., Van Der Smagt P., Cremers D., Brox T. Flownet: Learning optical flow with convolutional networks. Proceedings of the IEEE International Conference on Computer Vision.

[22] Zhu X., Xiong Y., Dai J., Yuan L., Wei Y. Deep feature flow for video recognition. Proceedings of the IEEE Conference on Computer Vision and Pattern Recognition.

[23] Wang S., Zhou Y., Yan J., Deng Z. Fully motion-aware network for video object detection. Proceedings of the European Conference on Computer Vision (ECCV).

[24] Newell A., Yang K., Deng J. Stacked hourglass networks for human pose estimation. Proceedings of the Computer Vision–ECCV 2016: 14th European Conference.

[25] Wang J., Sun K., Cheng T., Jiang B., Deng C., Zhao Y., Liu D., Mu Y., Tan M., Wang X. (2020). Deep high-resolution representation learning for visual recognition. IEEE Trans. Pattern Anal. Mach. Intell..

[26] Liu M., Zhu M. Mobile video object detection with temporally-aware feature maps. Proceedings of the IEEE Conference on Computer Vision and Pattern Recognition.

[27] Wang L., Tong Z., Ji B., Wu G. Tdn: Temporal difference networks for efficient action recognition. Proceedings of the IEEE/CVF Conference on Computer Vision and Pattern Recognition.

[28] Lin T.-Y., Goyal P., Girshick R., He K., Dollár P. Focal loss for dense object detection. Proceedings of the IEEE International Conference on Computer Vision.

[29] Dai J., Qi H., Xiong Y., Li Y., Zhang G., Hu H., Wei Y. Deformable convolutional networks. Proceedings of the IEEE International Conference on Computer Vision.

[30] Hu J., Shen L., Sun G. Squeeze-and-excitation networks. Proceedings of the IEEE Conference on Computer Vision and Pattern Recognition.

[31] Wang C.-Y., Bochkovskiy A., Liao H.-Y.M. YOLOv7: Trainable bag-of-freebies sets new state-of-the-art for real-time object detectors. Proceedings of the IEEE/CVF Conference on Computer Vision and Pattern Recognition.

[32] Reis D., Kupec J., Hong J., Daoudi A. (2023). Real-Time Flying Object Detection with YOLOv8. arXiv.

[33] Cai Z., Vasconcelos N. Cascade r-cnn: Delving into high quality object detection. Proceedings of the IEEE Conference on Computer Vision and Pattern Recognition.

[34] Ying X., Liu L., Wang Y., Li R., Chen N., Lin Z., Sheng W., Zhou S. Mapping Degeneration Meets Label Evolution: Learning Infrared Small Target Detection with Single Point Supervision. Proceedings of the IEEE/CVF Conference on Computer Vision and Pattern Recognition.

[35] Hu Z., Wang Y., Li P., Qin J., Xie H., Wei M. ISmallNet: Densely Nested Network with Label Decoupling for Infrared Small Target Detection. Proceedings of the ICASSP 2023—2023 IEEE International Conference on Acoustics, Speech and Signal Processing (ICASSP).

[36] Zhao Y., Shi Y., Wang Z. The improved YOLOV5 algorithm and its application in small target detection. Proceedings of the International Conference on Intelligent Robotics and Applications.

[37] Sunkara R., Luo T. No more strided convolutions or pooling: A new CNN building block for low-resolution images and small objects. Proceedings of the Joint European Conference on Machine Learning and Knowledge Discovery in Databases.

[38] Rivest J.-F., Fortin R. (1996). Detection of dim targets in digital infrared imagery by morphological image processing. Opt. Eng..

[39] Deshpande S.D., Er M.H., Venkateswarlu R., Chan P. Max-mean and max-median filters for detection of small targets. Proceedings of the Signal and Data Processing of Small Targets.

[40] Chen C.P., Li H., Wei Y., Xia T., Tang Y.Y. (2013). A local contrast method for small infrared target detection. IEEE Trans. Geosci. Remote Sens..

[41] Han J., Moradi S., Faramarzi I., Liu C., Zhang H., Zhao Q. (2019). A local contrast method for infrared small-target detection utilizing a tri-layer window. IEEE Geosci. Remote Sens. Lett..

[42] Gao C., Meng D., Yang Y., Wang Y., Zhou X., Hauptmann A.G. (2013). Infrared patch-image model for small target detection in a single image. IEEE Trans. Image Process..

[43] Dai Y., Wu Y. (2017). Reweighted infrared patch-tensor model with both nonlocal and local priors for single-frame small target detection. IEEE J. Sel. Top. Appl. Earth Obs. Remote Sens..

[44] Zhao M., Cheng L., Yang X., Feng P., Liu L., Wu N. (2019). TBC-Net: A real-time detector for infrared small target detection using semantic constraint. arXiv.

[45] Wang H., Zhou L., Wang L. Miss detection vs. false alarm: Adversarial learning for small object segmentation in infrared images. Proceedings of the IEEE/CVF International Conference on Computer Vision.

[46] Dai Y., Wu Y., Zhou F., Barnard K. Asymmetric contextual modulation for infrared small target detection. Proceedings of the IEEE/CVF Winter Conference on Applications of Computer Vision.

[47] Dai Y., Wu Y., Zhou F., Barnard K. (2021). Attentional local contrast networks for infrared small target detection. IEEE Trans. Geosci. Remote Sens..

